# Latent Rheumatism

**Published:** 1887-09

**Authors:** Charles Moore Jessop

**Affiliations:** Physician to the St. Pancras and Northern Dispensary


THE BRISTOL

Medico-Chirurgical Journal.

SEPTEMBER, 1887.

### LATENT RHEUMATISM.

BY

CHARLES MOORE JESSOP, M.R.C.P.,

*Physician to the St. Pancras and Northern Dispensary.*

An out-patient note-book may be made valuable when there is time and efficient assistance to record cases; but single-handed, and with a large number of patients, this is not always possible. Nevertheless, I venture to hope that the following imperfect notes, of two or three cases out of many, may be useful in drawing attention to one of the minor ailments of life, under cover of which there may be prolonged malaise, or formidable disease develop.

From text-books there is little to be learned on the subject of latent subacute rheumatism. The fullest description of the subject is found in Quain's *Dictionary*, and there only alluded to with reference to the growing pains of children.

The pains which young people, especially girls, complain of in the left breast are generally treated with scant consideration, being referred to indigestion, hysteria, or rheumatism, for which no particular treatment or examination is required, because there is no particular appearance or sign of active disease. It has often occurred to me that there was something of more serious import underlying this condition, and that the seat of discomfort was not duly examined from motives of delicacy; and, also, because in young girls or women both glands at a certain age take on a hyperæsthetic condition. In the cases to be detailed the pain was confined to the left breast, and had nothing to do with the gland, for when the gland was moved bodily there was no pain: pain was only elicited when digital pressure was employed between the ribs beneath the gland, chiefly below the apex-beat and to the left. Besides this pain there were also "fleeting pains" in one or more of the joints, so slight as to be forgotten until enquired for—even then not always remembered distinctly. The urine had not always been noticed, and might or might not be high-coloured. As a rule, no feverish condition was complained of, but generally constipation; and bruit de diable was always present. Occasionally palpitation and shortness of breath. Menstruation not always normal.

At Christmas, 1883, E. S., æt. 20, came to my house at Preston. She was naturally bright and active, and three months previously appeared to be in good health. But on this occasion she was dull and listless; her movements slow and languid, walking with care, soon out of breath, and exceedingly pale. Made no complaint of pain anywhere, but sat still and did nothing. The heart's sounds were muffled; there was a soft systolic bruit at the apex, with tenderness on pressure over the whole of the cardiac region. No abnormal dulness, and no friction sound. Slight dulness over the right apex, and feeble respiratory murmur. No cough. Bruit de diable loud. An œdematous condition of both ankles and legs, chiefly the left. There were tenderness and hardness in the course of the left femoral vein. Pain on pressure over the right hip-joint, with some fulness; afterwards in the left hip-joint. The right apex, cardiac region and hip-joints were painted with iodine liniment every second or third day according to the extent of tenderness. Internally dilute nitro-muriatic acid in twenty-drop doses, with tincture of iron in fifteen-drop doses, were prescribed three times daily. This was varied from time to time, so that the stomach should not be wearied by one form of drug. But the external application was persisted in so long as there was any tenderness or swelling. The femoral vein was left to take care of itself; as her health improved, the tenderness and hardness gradually disappeared. At the end of two months she was able to run up and down stairs without reproducing the murmur, when she was transferred to Bournemouth, where she completed her recovery.

This young lady's case perplexed me considerably at first, for the idea of latent subacute rheumatism was not present to the mind. The above detail was not unfolded all at once: the heart and vessels first engaged attention; later on, the hip-joints; then, gradually, the pathological relation between the heart and hip-joints was established.

Rheumatism is characterised by inflammation of the fibrous structures, and when it is latent "the joints are so slightly affected that its signs and symptoms may be entirely overlooked." Pain and tenderness in children are often disregarded, or called "growing pains," or ascribed to "a cold." The subjects of these "fleeting pains" and cardiac tenderness are not confined to children, but extend to others of older growth. In the absence of the well-known symptoms of acute rheumatism, there is a latent form which, if recognised and treated, leads to ease, comfort, and recovery; if neglected, may lead to long-continued discomfort, for which the doctor obtains no credit.

Women are more subject to rheumatic diseases of the heart than men; this may be the result of imperfect nutrition, induced by the action of stays, which by compressing the lower ribs prevent the gentle rise and fall of abdominal viscera, especially expansion of the liver and spleen during digestion, essential to a free performance of their duties, and prohibit the heart's descent of one inch and a half eighteen times in a minute, to say nothing of the structural injury inflicted on the liver and stomach. This impediment to the heart's free movement has, it appears to me, much to do with the pain situated near the heart's apex in some cases. It is discoverable only by digital pressure or on application of an intercostal stethoscope. Probably this pain arises from a hyperæmic condition of that portion of the pericardium attached to the central tendon of the diaphragm, through abnormal approximation of the visceral and parietal layers due to imperfect descent of the diaphragm in respiration. This pain and tenderness, then, might more properly be referred to pericarditis, which in many cases is so mild a disorder that it is attended with few or no symptoms of importance. "In the mildest form pericarditis often escapes detection, or is recognised only by the accidental discovery of pericardial friction; in most such cases, however, there is some time or other slight præcordial pain or uneasiness, together with extension of cardiac dulness and febrile disturbance. Most cases of what are termed 'latent' and 'dry' pericarditis belong to this group." The symptoms of rheumatic pericarditis are "dyspnœa, palpitation, and a sense of oppression increased by pressure in the intercostal spaces." "Muscular rheumatism also attacks the heart, causing violent palpitation."

The following case of a dispensary patient, following her daily occupation in an upholsterer's shop, combines an interesting series of events which illustrates most of the foregoing extracts from well-known text-books :—

E. R., æt. 19, 5th Feb., 1887, a fresh-complexioned, stout, healthy-looking girl, pale, complained of pain over the left breast, a fulness after meals with some eructation; of pain and violent palpitation at night. Has had "fleeting pains" in several joints, at present in her elbows and forehead. There is some heaving in the cardiac region, with tumultuous action of the heart, shortness of breath on exertion, and pain on pressure in the intercostal spaces, mostly in the 5th and 6th interspaces, extending to the outer side of the breast. Slight bruit de diable, clean tongue, slight constipation. Liniment of iodine was applied once a week, and a mixture of nitro-muriatic acid diluted, with digitalis and quassia. 19th Feb. she was much better; able to scrub out a room and walk upstairs without distress. Heart's action quieter, and pain limited to one small spot. Iron tincture was added to the mixture. 26th Feb. had lost all pain in her joints, but complained of tightness over the cardiac region. The heart is still tumultuous. On the 5th March the digitalis was omitted from the mixture. On the 8th March the tightness had increased, but the heart's action was quieter, and there was considerable swelling of the 6th and 7th intercostal cartilages at their junction with the ensiform cartilage—a distinct oval prominence about an inch and a half long and not quite half an inch broad. It was not tender. It was painted with iodine. On the 12th March this swelling had somewhat subsided, but she now complained of oppressive tightness over the manubrium sterni. There was no pitting, no redness, but the skin was not freely movable. On the 19th March the cartilaginous swelling had further decreased, but the tightness over the manubrium had increased. She complained of pain and oppression mostly between 3 and 5 p.m., or for longer; and it was especially worse if the workroom became hot. There was no tenderness, but a diffused swelling and immobility of the skin. Mixture was continued. On the 26th March, the heart's action was normal; the tight area had decreased, the cartilaginous swelling was going down. The painting with iodine was steadily continued. 2nd April. Had felt pretty well during the past week: only complained of the pain and sensation of tightness twice. 9th April. Tumefaction over the sternum disappearing, and felt the tight feeling only once. The lower swelling almost imperceptible. On the 16th April she was fairly well, and ceased to attend. On the 20th May she wrote to say she was not able to attend, but was a little better; so it is presumed she had not entirely recovered, or probably suffered from slight relapse.

Mrs. R., æt. 30, pale and thin, on the 14th May complained of pain in her joints. She had some feverish symptoms and thirst, with pain on pressure beneath the left breast. No palpitation. The same mixture and local application were employed. On the 17th May she had improved, but had cartilaginous swelling of the 6th,

7th, and 8th ribs at their junction with the ensiform cartilage, also pain in the right knee and toes. On the 21st May she was improving. On the 28th she did not appear; so it may be presumed she is progressing favourably.

She made a good recovery.

E. T., æt. 27, 1st March, 1887, a cook, stout and of strumous habit, pale, had pain in joints and pain in the cardiac region. On pressure in the intercostal spaces, there was considerable tenderness extending from the left border of the sternum beyond the nipple-line. She had scanty and irregular periods of menstruation, constipation, and bruit de diable. She had inhabited a room over the water-tank of the house, and many former occupiers had been unwell. Last year she was under treatment for anæmia and had recovered, living in the same situation, but now occupying a better bedroom. A mixture of iron, acids, Epsom salts and quassia, with cod oil, were prescribed, and weekly painting with iodine. On the 5th April she was considerably improved in all respects. The cardiac pain had not entirely disappeared. The local application was used once a fortnight. On the 24th May she had lost all cardiac tenderness, and felt free from pain and discomfort: to continue iron, arsenic, and chiretta mixture, with cod oil, for another fortnight.

A. M., æt. 16, a kitchen-maid, stout, fresh-complexioned girl. Gums and fauces pale, loud bruit de diable. "Fleeting pains" in her joints, cardiac pain over area of pulmonary artery; this gradually disappeared, and was transferred to the 5th and 6th interspaces. Suffered from constipation. No appetite; menstrual flow pale. Tenderness over 1st and 2nd dorsal vertebræ. Ordered to take acid, iron, and quassia mixture after meals, with aloes and iron pill at bedtime. On the 10th May was much improved: to continue mixture, and take a morning aperient of scruple doses of compound jalap powder and sulphur. Locally, the iodine liniment as before. On the 24th May she had nearly lost the cardiac tenderness and that over the dorsal vertebræ. Going for a month into the country.

She returned from the country with incipient varicose vein in the left leg, a return of pain in the spine and cardiac region. Complained of "fainting fits" in her household work. Being a good-looking girl, though stout, she aspired to slimness of waist: the "fits" ceased when the stays were worn loose; she speedily recovered.

A stout, active lady, æt. 54, fell downstairs last September. Six weeks later suffered from inability to take carriage exercise, owing to extreme tenderness of the whole spinal column. She so far recovered this, that in March she left London for the country. On several occasions she had complained of a rheumatic affection of the joints, "fleeting pains," and latterly of puffiness of the face and swelling of the left jaw. Just before leaving, the heart's sounds were found to be muffled at the apex. She looked pale, and generally out of health, though she had been taking various tonics. Her brother, a medical man, was communicated with; he found "the percussion note normal all over the cardiac area; the apex-beat in the right position. I found, as you say, the sounds rather muffled and distant at the base; but at the apex they were very clear. Both sounds otherwise were normal; and there is certainly no evidence of valvular mischief. The pulse too is regular and of good volume, 76. She complains very much of pain over the occipito-parietal suture, which, I should imagine, is due to the great nervous shock she must have received in that fall; and I should consider the muffled heart-sound is due to the same cause. Her tongue is very flabby, and indicative of want of tone." Prescribed acid, nux vomica, digitalis, and quassia. She improved greatly, and returned to London at the end of three weeks. The heart's sounds were found to be stronger at the apex, and the general aspect improved. She continued the mixture she had been taking for a week longer. On digital pressure below the apex-beat, and over the left and outer side of the cardiac region, pain was elicited; to this, liniment of iodine was applied once a week. Tenderness has slowly decreased; only a small tender spot remains. Within ten days of her return, puffiness of the face and swelling of the jaw re-appeared for a couple of days. At present, the heart's sounds are full-toned and regular. Dyspnœa on prolonged exertion.

Had the cardiac tenderness been recognised in this case there would probably have been no relapse, and a lingering malaise avoided. Her improved health is attributable solely to the local treatment, which is not yet omitted.

The local treatment was continued fortnightly for two months longer. She is now well, and can walk uphill without discomfort.

I have ventured to record these few cases, recently under treatment, because the persons are fresh in the memory, for the purpose of drawing attention to careful digital exploration of the intercostal spaces in the cardiac neighbourhood, and to show the value of local treatment in cases which appear to be trifling. There was no hysterical tendency in any of the patients; and to guard against sympathetic influence, comparison was made with the opposite side. All the cases were, what would be termed, "anæmic," and would be treated by restoratives; but an endeavour has been made to show that these agents will not of themselves, unless supplemented, suffice to produce a restoration to health in rheumatic anæmia or latent subacute rheumatism, a condition which has to be sought out.

Before concluding this communication, I may perhaps be permitted to mention the value of free painting with liniment of iodine in acute rheumatic fever. A schoolboy, of 14 years, had a severe attack, with the usual high temperature, sweats, and so forth. The first day he was seen, the heart's sounds were found *slightly* muffled. The whole of the cardiac area was painted; the following day all muffling had disappeared. Every joint in his body in succession was attacked; each was painted with iodine on the occurrence of pain or redness. The painting was repeated in many instances, and the cardiac areas required repeated attention. The other treatment was with bark and acids, and finally iron. The case was extremely severe; but he made a good recovery, and without the slightest damage to the heart. It is unnecessary to point out the value of this counter-irritation on the local and general circulation, nor yet on the reflex action through the brain and cord centres, by which a change is induced in the nutrition of the tissues.

